# Is Peri-Operative Isolated Systolic Hypertension (ISH) a Cardiac Risk Factor?

**DOI:** 10.2174/157340308783565410

**Published:** 2008-02

**Authors:** Ashraf Fayad, Homer Yang

**Affiliations:** Department of Anesthesiology and Perioperative Medicine, University of Ottawa, 1053 Carling Ave. (B3), The Ottawa Hospital, Ottawa, Ontario, Canada, K1Y 4E9

**Keywords:** Isolated systolic hypertension, cardiovascular risk factors, perioperative.

## Abstract

We are presenting a review of Isolated Systolic Hypertension (ISH) as a cardiovascular risk factor with emphasis on the perioperative period.

Isolated systolic hypertension is associated with aging and is the most frequent subtype (65%) among patients with uncontrolled hypertension. ISH is strongly associated with increased risks of cardiac and cerebrovascular events exceeding those in comparably aged individuals with diastolic hypertension. Patients with ISH show an increase in left ventricular (LV) mass and an increase in the prevalence of left ventricular hypertrophy (LVH). These LV changes increase cardiovascular events and frequently lead to diastolic dysfunction (DD). Treatment to reduce elevated systolic blood pressure has been shown to reduce the risk of cardiovascular events.

In the perioperative setting, essential hypertension has not been found to be a significant risk factor for cardiac complications. Most of the studies were based on the definition of essential hypertension and underpowered in sample size. The significance of perioperative ISH, however, is not well studied, partly due to its recognition only fairly recently as a cardiovascular risk factor in the non-surgical setting, and partly due to the evolving definition of ISH.

Perioperative cardiac complications remain a significant problem to the healthcare system and to the patient. Although the incidence of perioperative cardiac complications is prominent in high-risk patients as defined by the Revised Cardiac Risk Index (RCRI), the bulk of the cardiac complications actually occur in low-risk group. Currently, little understanding exists on the occurrence of perioperative cardiac complications in low- risk patients. A factor such as ISH, with its known pathophysiological changes, is a potential perioperative risk factor.

We believe ISH is an under-recognized perioperative risk factor and deserves further studying. Our research group has recently been funded by the Heart Stroke Foundation (HSF) to examine ISH as a perioperative risk factor (PROMISE Study).

## I) ISOLATED SYSTOLIC HYPERTENSION AS A CARDIOVASCULAR RISK FACTOR

### Background

Isolated Systolic Hypertension (ISH) is increasingly recognized as a cardiovascular risk [1,2]. Several studies have shown the strong association between ISH and stroke, cardiovascular morbidity and mortality [3-5]. Data from Multiple Risk Factor Intervention Trial (MRFIT) demonstrated a continuous and graded influence of systolic blood pressure (SBP) on coronary heart disease mortality [6]. The study found that the greatest number of deaths is seen in patients with SBP between 140 and 149 mmHg. However, the highest risk of death was found in patients with SBP > 180 mmHg (Fig. (**1**)). It also found that SBP was a stronger predictor of outcome than diastolic blood pressure (DBP). The JNC-VII report confirms the close association between ISH and cardiovascular events [7]. It also highlights that; SBP greater than 140 mm Hg is a more important cardiovascular risk factor than an elevated DBP. The risk of cardiovascular disease begins at 115/75 and doubles with each increment of 20/10 mmHg.

The continuous graded influence of high blood pressure on cardiovascular disease (CVD) risk was also found in the meta-analysis of Prospective Studies Collaboration [8]. It involved 61 prospective studies with 1 million participants. There were 56,000 vascular deaths related to high blood pressure, without any indication of a threshold down to 115/75 mmHg. The meta-analysis clearly demonstrated the higher incidence of ischemic cardiac events and strokes with elevated SBP (Figs. (**2** & **3**)). Masley *et al.* [9], in a recent follow up study, evaluated the association between blood pressure and the risk of developing CVD in 4,008 elderly patients. The study found that elevated SBP was strongly associated with developing future CVD events (P=0.0001); the relationship is linear, graded and holds for ages above and below 75 years old. It also demonstrated that the frequency of CVD was the lowest with SBP< 120 mmHg while elevated DBP didn’t seem to be a major factor for future CVD events. Other clinical trials, along with MRFIT, have gone a step further to demonstrate that an excess risk of cardiovascular diseases exists not only in patients with ISH but also in pre-hypertensive subjects [10-12].

### Definition and Classification of ISH:

The definition and classification of ISH have undergone several iterations. ISH is currently defined as SBP > 140 mmHg with DBP < 90 mmHg, and not secondary to another disorder in individuals aged 18 and older. This is based on the average of 2 or more seated BP readings on each of 2 or more office visits. The definition is adopted by the Seventh Report of the Joint National Committee (JNC-VII) on Prevention, Detection, Evaluation, and Treatment of High Blood pressure [7]. The main changes in the classification from the previous one (JNC VI) were the development of pre-hypertension as a new classification category and the combining of stage 2 and stage 3 hypertension into a single stage 2 category (Table **1**).

Pre-hypertension (SBP of 120 – 130 mmHg) is not a disease category. Rather it is defined in order to identify individuals at risk of developing hypertension. They are not candidates for treatment but will require health-promoting lifestyle modifications to prevent cardiovascular disease. Classification of the JNC VII does not stratify hypertensive patients by the presence or absence of risk factors. Also, it does not stratify them by the presence or absence of target organ damage. Therefore, the JNC VII treatment recommendation does not take into consideration if either or both are present. However, it recommends that all individuals with stage 1 or 2 hypertension be treated.

### Epidemiology of ISH

The prevalence of ISH increases with age, and it accounts for substantial proportion of hypertensive patients. It is the most frequent subtype of hypertension in the US [13]. The rise in SBP continues throughout life, in contrast to DBP, which rises until 50 years old, tends to level off over the next decade, and may remain the same or decline later in life [14]. All of these studies suggest that age 50 years is a useful cut point to dichotomize arbitrarily hypertensive individuals into two groups for the purpose of classifying hypertension by subtype (Fig. (**4**)). 

In the National Health and Nutrition Examination survey (NHANES III), 42.7 million adult Americans were identified as hypertensive, which represented 24% of the adjusted adult US population [15]. Over 65% of the uncontrolled or untreated hypertensive population had ISH. Participants ≥ 50 years of age comprised three fourths of all hypertensive subjects. The older group was predominantly female (58%), whereas the younger group was predominantly male (62.5%). The predominant hypertensive subtype was ISH (79.8%) in the older group (75% stage 1 and 25% stage 2 or higher) and isolated diastolic hypertension (42.8%) in the younger group (98% stage 1 and 2% stage 2 or higher). Prevalence of ISH may be higher in some populations. A study looked at the characteristics of ISH in bi-racial Evans County, Georgia, from 1967 to 69. It found that the prevalence of ISH was higher in females and in the black population [16].

The Canadian Heart Health Survey (CHHS) showed that the overall prevalence of ISH was 8.1% and the prevalence of untreated ISH was 6.4% [17]. Figures from Australia suggested an ISH prevalence of 8.6% in patient ≥ 60 years old [18]. Supporting data from the JNC-VII noted that although 59% of hypertensive patients were receiving treatment, only 34% had blood pressure levels below 140/90 [7].

In the WHO report, sub optimal BP (> 115 mm Hg SBP) is responsible for 62% of cerebrovascular disease and 49% of ischemic heart disease [19]. However, the WHO guidelines set a lower threshold than those advocated by JNC-VII. Acceptance of the WHO threshold results in 60% of the population being classified as hypertensive [20]. Taking into consideration the current definition of ISH, it does not only become the most common subtype of hypertension worldwide but also the most prevalent untreated hypertension among people over 60 years of age [21]. In a recent European study, the prevalence of ISH in patients over 55 years was found to range between 20.3% in primary care patients and 35% in the community, using mean values of both systolic and diastolic blood pressure [22]. The study included over 1,000 participants with all blood pressure measurements recorded in accordance with the standards of the JNC VI. In the years to come, the number of elderly among the population is expected to rise rapidly and clearly ISH prevalence will be an even more important issue.

### Pathophysiology of ISH and its Cardiac Sequel

Data from clinical studies indicate that ISH is a distinct syndrome with pathophysiological changes different from those in essential hypertension [23]. The main feature of ISH is a decreased distensibility of the aorta and large arteries with subsequent cardiovascular events [24,25]. Mechanical changes in the arterial walls such as atherosclerosis and the gradual loss of aortic distensibility, occurring over the course of the ISH, was thought to be a natural event of the aging process [26]. Framingham heart study has also demonstrated an increase in arterial stiffness due to aging or pathology and a decrease in arterial distensibility [27]. Though other studies have questioned the reduction in distensibility as a natural consequence of aging [23,28], the recent Framin-gham offspring study involved healthy individuals demonstrated age-related increase in aortic stiffness with little change in peripheral arteries [29]. It is not known whether the decreased distensibility or the elevation of systolic blood pressure occur first. Nevertheless, the elevation of SBP increases the sheer stress and fatigue on arterial walls, accelerating arterial damage, exacerbating the arterial changes and atherosclerosis, creating a vicious cycle of plaque formation and rupture, further blood vessel damage, and loss of arterial distensibility. Pathology examination of the aorta in elderly patients with ISH reveals thickening of the media due to the accumulation of the collagenous fibers and calcium [30]. The resultant decrease in the arterial compliance results in higher SBP and pulse-wave velocity as the large arteries become less able to reduce the pressure generated by the left ventricle (LV) by means of distension (cushion function). While increases in peripheral resistance will cause elevations in DBP, the loss of large-artery elasticity, but not in the smaller arteries (resistant arterioles) as in diastolic hypertension [31], leads to less blood and therefore less potential energy being stored for forward flow during ventricular diastole. Thus for a given heart rate and peripheral resistance, diastolic pressure is low. In addition, ISH impairs conduit artery endothelial function whereas diastolic hypertension induces endothelial dysfunction in resistance arteries in the elderly [32].

The heart adapts to the ensuing increase in wall tension by hypertrophy and increased myocardial contraction time. Although these adaptations seem to preserve systolic function, diastolic impairment becomes apparent as LV compliance and early diastolic filling decline  [33,34]. As a result, ISH patients demonstrate an increase in LV mass and a high prevalence of LV hypertrophy (LVH) compared with age-matched normotensive individuals [35,36]. When compared to diastolic hypertension, the prevalence of LVH was higher in ISH patients despite lower mean blood pressure [37]. These LV changes occur over time and usually carry prognostic significance [38,39]. Studies have shown that LVH is associated with an increase in cardiovascular events (stroke, ischemic heart disease and heart failure) [39,40]. In a recent study on coronary blood flow in normal subjects versus patients with LVH, there was a significant reduction of the peak coronary flow during diastole in LVH patients [41]. In addition, the correlation between hypertension, LVH and left atrium (LA) size is well documented [42]. Data suggest that LA size is an important factor in the development of atrial fibrillation and other cardiovascular events [43,44].

The presence of LVH in ISH patients frequently leads to diastolic dysfunction (DD) [45,46]. Though DD may be seen in the elderly without high blood pressure or increased LV mass [47], a higher prevalence of DD was seen in patients with ISH stage 1 (systolic pressures between 140-159 mm Hg) without any impairment of systolic function [48]. Left ventricular end diastolic pressure (LVEDP) is an important factor that determines oxygen supply to the myocardium, as it is the pressure, which is exerted directly on the subendocardium. The amount of flow entering the coronary circulation during diastole is the result of the pressure gradient between the epicardial coronary artery and the subendocardium. Elevation of the LVEDP as in DD patients can reduce this gradient significantly, and hence, decreasing the coronary blood flow and myocardial perfusion [49]. The oxygen cost of “pressure work” is greater than “volume work”, with the area-under-the-curve for LV pressure closely correlating with myocardial oxygen demand [50]. In hypertensive patients, free of coronary artery stenosis, DD may be a determinant in the impairment of the coronary microvascular vasodilation capacity or a marker of silent ischemia involving the microvascular circulation [51]. In essence, the combination of lower diastolic blood pressure, LVH, LA, and DD in ISH may compromise subendocardial myocardial perfusion.

### ISH and Cardiovascular Events

For decades, despite the focus on DBP, many investigators know that ISH is a cardiovascular risk factor, frequently seen in the elderly [21,52-55]. Recent studies however, have renewed interest in ISH and accumulating evidence warrants greater attention to the importance of SBP as a major factor for CVD.

In a cardiovascular health study on hypertensive predictors of cardiovascular events (myocardial infarction, stroke and mortality), SBP was the single best predictor of cardiovascular events [56]. After adjusting for myocardial infarction, a one-standard deviation change in SBP, DBP, and pulse pressure was associated with hazard ratios (95% confidence intervals) of 1.24 (1.15-1.35), 1.13 (1.04-1.22), and 1.21 (1.12-1.31), respectively. For stroke, the hazard ratios (95% confidence intervals) were 1.34 (1.21-1.47) with SBP, 1.29 (1.17-1.42) with DBP, and 1.21 (1.10-1.34) with pulse pressure. The study involved 5,888 participants recruited from four US centers aged 65 years and older. The average follow up in the study was 6.7 years with a linear association between blood pressure level and cardiovascular disease risk.

Further supporting data comes from the analysis of the Framingham 30-year study. It demonstrated that SBP is a strong and a consistent predictor of coronary artery disease over several age strata [57]. The MRFIT trial with its initial 361,662 participants also provides evidence of the close relationship between the level of SBP and coronary heart disease. The age of the MRFIT trial participants ranged between 36-57 years old when they were first screened with 3 successive screenings and frequent follow up [58-60].

Studies from Europe have also demonstrated a positive continuous, graded, and independent association for both total (P<. 001) and cardiovascular (P< .001) mortality with SBP but not DBP. Blood pressure was linearly related to the risk of stroke in untreated patients [61] but, in patients with treated hypertension, the relationship of both SBP and DBP to stroke was J-shaped with increased risk at high and low levels of blood pressure [62]. The risk of first-ever stroke was associated with hypertension (relative risk, 1.6; 95% CI, 1.2 to 2.0) and with isolated ISH (relative risk, 1.7; 95% CI, 1.1 to 2.6). In a recent study this J shape pattern was not seen in general population or in individuals without chronic renal insufficiency  [63].

A study from China examined ISH, isolated diastolic hypertension and systolic-diastolic hypertension as predictors of stroke [64]. The relative risks of stroke with the subtypes of hypertension, compared with normotensives, were estimated using the Cox model. After adjustments for the confounders, the prevalence of hypertension was: ISH 7.1%, systolic-diastolic hypertension 18.4%, isolated diastolic hypertension 6.7%, and controlled hypertension 3.9%. The follow up study included a total of 233,437 patients. There were 1,107 stroke (614 ischemic and 451 hemorrhagic events and 42 unclassified). The hazard ratio and 95% CI was 2.96 (2.49 to 3.52) for all stroke. Both ISH and isolated diastolic hypertension were independent predictors of stroke. Patients with systolic-diastolic hypertension however, were at the highest risk of stroke and should be treated aggressively.

Beside its cardiovascular risk, high blood pressure is regarded as a major risk factor for the development of renal insufficiency [65]. When both SBP and DBP variables were considered, the estimated risk of chronic renal insufficiency was higher with elevated SBP [66]. 

### Treatment of ISH and the reduction of Cardiovascular Events

Treatment to reduce elevated systolic blood pressure to less than 140 mmHg has been shown to reduce significantly the risk of cardiovascular events [67-70]. The Systolic Hypertension in the Elderly program (SHEP) [67] was the first large-scale trial to document a benefit from treatment of ISH. The 4,736 patients were enrolled in a double blind, randomized, placebo-controlled study. Patients in this trial were treated with chlorthilidone ± atenolol to achieve SBP < 160 mmHg. The primary end point was the number of fatal and nonfatal strokes with the cardiac events as the secondary end points. Though the definition of ISH in the trial was outdated, there was a 36% reduction in stroke risk (P= <0.003), 25% reduction in coronary heart disease (P=<0.05) and 32% fall in total cardiovascular disease evens (P=<0.05) in the active treatment group compared to the placebo group.

Encouraging results were also obtained from the Systolic Hypertension in Europe (Syst-Euro) trial [68,71,72]. In the trial, 4,695 patients were randomized to receive the long-acting dihydropyridine calcium channel blocker nitrendipine or a placebo. If BP was not controlled, enalapril, hydrochlorthiazide, or both were added. The primary end point was fatal and nonfatal strokes. Similar results to the SHEP were obtained. There was a 43% (P= 0.003) reduction of fatal or non-fatal stroke in patients treated actively to achieve SBP < 150 mmHg. As in the SHEP study, total mortality was not reduced significantly by active treatment. The extended follow-up study (Syst-Eur2) [73] of the trial obtained similar results.

A reduction in the incidence of stroke and other cardiovascular complications was also achieved in the Systolic hypertension in China (Syst-China) [74] and the Shanghai Trial of Nifedipine in the Elderly (STONE) in the treatment groups [75]. The Syst-China follow up study did not demonstrate a significant overall influence on the active treatment group but there were clear benefits of treatment in diabetic and non-smoker patients [76]. Further, the meta-analysis of ISH outcome trials showed that the treatment of ISH confers a protective effect against stroke and coronary heart disease risk [77].

Different drug therapies have been suggested to treat systolic blood pressure. These included the use of diuretics, beta-blockers, calcium channel blockers and angiotensin converting enzyme inhibitors (ACEI). The JNC-VII (Table **1**) has also recommended the use of a single antihypertensive therapy or a combination of two or more for stage 1 and stage 2 hypertension. The choice of the antihypertensive usually takes into consideration the type and subtype of hypertension, the severity, and the co-morbidities. In addition, when prescribing an antihypertensive medication, clinicians should also be aware of the patient’s compliance to treatment.

A recent study suggested that caution should be exercised on lowering blood pressure less than 140/90 in patients over 80 years old [78]. In another study, lowering DBP (< 65-70 mmHg) in the course of treating ISH may be associated with an increase risk of stroke and other cardiovascular events [69].

## II) ISOLATED SYSTOLIC HYPERTENSION AS A PERIOPERATIVE CARDIOVASCULAR RISK FACTOR

### Recognition of the Problem

Sprague, in 1929, was the first to examine the relationship between hypertension and perioperative cardiac risks. In his report, he described a series of 75 hypertensive patients of whom 1/3 died in the perioperative period [79]. Prys-Roberts conducted a series of “studies of anesthesia in relation to hypertension” [80-83]. Intraoperative hemodynamic instability was found to be the major cardiovascular event in uncontrolled hypertensive patients. The first of his studies examined 29 patients undergoing elective non-cardiac surgery [80]: there were 7 normotensive, 7 untreated and 15 patients on antihypertensive medications. Labile BP associated with myocardial ischemia was noted during induction of anesthesia and during the intraoperative course in the untreated group. The subsequent study with 36 hypertensive patients assessed the electrocardiographic and hemodynamic responses to induction of anesthesia, laryngoscopy and endotracheal intubation [81]. There were 16 untreated patients and 20 on antihypertensive medications. Treated and untreated patients were divided into 5 groups based on the type of anesthesia induction (thiopentone, neuroleptanalgesia, propanidid, diazepam and methohexitone). Surprisingly, the study found that patients on antihypertensive therapy with well-controlled blood pressure were equally prone to develop hemodynamic instability and myocardial ischemia. However, close analysis of the preoperative BP values, using the JNC-VII definition of hypertension, identifies 13 ISH patients who were considered controlled hypertensive or normotensive patients. The conclusion was that patients with uncontrolled or poorly controlled hypertension, especially with DBP above 110 mmHg, should not undergo elective surgery until their blood pressure is controlled. The conclusion was not based on hard outcomes in perioperative cardiac complications but on the perioperative hemodynamic instability. Goldman and Caldera, in 1979, compared 431 normotensive patients with 248 hypertensive patients [84]. There was a higher incidence of cardiovascular complications in the hypertensive group but it didn’t reach statistical significance. In essence, there was no indication of an increased risk of cardiovascular complications in the perioperative period, regardless of DBP. There was no reference to ISH. Other studies on perioperative risk factors supported the findings by Goldman and concluded that hypertension alone was a minor risk factor [85-87].

In a multicenter prospective study of 17,201 patients undergoing elective surgery, Forrest *et al.* [88] found 17 significant predictors of severe adverse cardiovascular events (deaths, cardiovascular outcome and respiratory outcome). Preoperative hypertension was found to be associated with an increased risk of perioperative bradycardia, tachycardia and hypertension. In another, 17,638 consecutive day-case surgical patients were examined to develop mathematical models to estimate the risk of perioperative adverse events in patients with pre-existing conditions [89]. Hypertension was found to predict the occurrence of any intraoperative event and intraoperative cardiovascular events. Shackelford *et al.* [90] evaluated Goldman and New York Heart Association cardiac risk index values as risk factors for perioperative cardiac morbidity in patients undergoing major vaginal surgery. It was a retrospective analysis of 406 patients with 8 patients having perioperative cardiac morbidity. The Goldman cardiac risk index and the New York Heart Association functional classification of heart disease were not significant indicators of perioperative cardiac morbidity in this group of patients. However, hypertension and ischemic heart disease were found to be risk factors for perioperative cardiac morbidity in a postmenopausal subgroup of these patients. None of the above studies had any reference to ISH or SBP.

Although well conducted at the time, we believe that these early studies failed to recognize ISH as a perioperative risk factor for the following reasons. First, ISH was recognized as a cardiovascular risk factor only in recent years. For decades, ISH was not deemed to be strongly associated with the same sequelae as in diastolic or combined hypertension [67,91,92]. Hence, the focus for perioperative studies has been mainly on DBP. Second, the definition of ISH and the emphasis on its treatment were established only recently at the JNC VI [93]. Third, many patients with high systolic blood pressure were not considered previously to be hypertensive as the earlier definition of ISH, utilized in most of the literature, was SBP > 160 mmHg. This led to a significant proportion of ISH patients being missed from the old perioperative hypertensive studies. Fourth, the sample sizes of previous perioperative hypertensive studies were too small to draw firm conclusions on perioperative hard outcomes without type II errors.

Howell *et al.* examined the role of a number of risk factors in the development of silent ischemia after general anesthesia for general and vascular surgery in 183 patients [94]. Patients were monitored pre- and postoperatively for ischemia using a Holter electrocardiography monitor. A history of hypertension, indicated by treatment with antihypertensive drugs, was associated with increased risk (odds ratio: 2.58, 95% confidence intervals: 1.12-5.96). A linear trend was found for risk associated with increasing admission systolic blood pressure (odds ratio: 1.20 for each 10-mmHg increase in systolic pressure, 95% confidence intervals: 1.01-1.42). However, the study did not specifically examined ISH as a perioperative predictor.

Also, Howell and co-workers examined the association between perioperative hypertension and cardiovascular complications including clinical trials, systematic reviews and meta-analysis [95-98]. There was an increase in the incidence of postoperative myocardial ischemia and an increase in cardiovascular death within 30 days of elective surgery  [95-97]. The recent meta-analysis of 30 observational studies involving 13,671 patients [99] has looked at hypertension and major perioperative cardiovascular outcomes. Major outcome complications were identified as cardiovascular death, myocardial infarction, new or more severe angina, heart failure, life threatening arrhythmias, and cerebrovascular accident. It demonstrated an odds ratio of 1.35 (1.17 – 1.56), which is not clinically significant. The study represents the most complete summation of evidence to-date; nevertheless, the definition for hypertension used by most of the studies in the meta-analysis predated the definition for ISH. The authors also found significant heterogeneity in the meta-analysis and attempted to isolate the source with a number of sensitivity analyses without success.

The only study looking at the association between ISH and postoperative cardiovascular events was carried out in the open-heart surgery patients [100]. It was a multicenter prospective observational study involved 2417 patients scheduled for elective CABG. The postoperative adverse outcome variables were congestive heart failure, renal insufficiency, central nervous system dysfunction and death. Of the 2069 that qualified for analysis, 612 had ISH. Among the 612 patients with systolic hypertension 52% had SBP between 140-150 mmHg. Most patients were receiving some type of antihypertensive medications and their mean age was 65 years with Caucasian majority. ISH combined with other risked factor was associated with 40% increase in likelihood of perioperative cardiovascular morbidity. This increase remains present regardless of antihypertensive medications, anesthetic techniques, and other perioperative cardiovascular risk factors. The prediction power of ISH was a 30% increase in the likelihood of an adverse outcome (odd ratio 1.3; 95% CI, 1.1-1.6). This study obviously provides evidence that ISH increases the risk of perioperative cardiovascular complications in patients undergoing CABG surgery. However, a limitation of this study was reliance on a single preoperative measurement of blood pressure.

Although the ACC/AHA guidelines do not identify hypertension as an independent risk factor for perioperative cardiovascular complications [80], it reflects more of the limitations of existing studies.

### Prevalence of the Perioperative ISH

The prevalence of perioperative ISH in non-cardiac surgery remains unclear. While it is unknown if the same percentage of ISH in the community presents for surgery, the proportion of untreated perioperative ISH is also unknown. In patients undergoing coronary artery bypass grafting (CABG), the prevalence of perioperative ISH was found to be 29.6% [100]. ISH is particularly prevalent in elderly patients and with the aging population, we will experience an increase in the prevalence [101]. By 2030, it is estimated that 20% of the population will be aged 65 and older with a significant increase in the number of surgical procedures among the elderly [102,103].

### Pathophysiology of ISH and the Perioperative Period

A study on 118 patients with uncomplicated mild hypertension using echocardiography found that concentric LV geometry was found mainly in untreated patients. This resulted in the identification of a high-risk subgroup with concentric remodeling, which had not been recognized or treated [104]. Although conducted in a non-surgical setting, such a study highlights the fact that physiological and anatomical adaptations may be more complex than merely accepting all uncomplicated hypertensive patients being the same. In the perioperative period after major non-cardiac surgery, the physiological and stress hormonal responses add another layer of complexity.

The development of LVH and DD are common in ISH patients [37,45]. Diastolic dysfunction in the aging heart is increasingly recognized as a cause of heart failure  [105-108]. History of heart failure is an important predictor postoperative adverse cardiac outcome and in hospital mortality in the elderly  [109,110]. Previous studies have shown that the pre-operative DD is present in 30 – 70% of cardiac surgical patients and has been associated with difficult weaning from cardiopulmonary bypass, the need for more frequent pharmacological support, and increased morbidity  [111,112]. In addition, preoperative DD was found to be a significant marker of in-hospital mortality after cardiac surgery and is independent of systolic function or the type of cardiac procedure [113]. Intraoperative acute DD is a new entity, which was observed by our research group during thoracoabdominal aortic aneurysm surgery [114]. In that study, postoperative myocardial infarction was significantly higher in patients who developed intraoperative acute DD. Numerous studies have examined diastolic dysfunction in the non-surgical setting and found a strong evidence to show that abnormalities of diastolic filling can determine the clinical status and the prognosis of the patients [115-117].

During the perioperative period, stress hormones and catecholamine surges are well reported [118-121]. Sympathetic activation during the induction of anesthesia can cause the blood pressure to rise by 20 - 30 mmHg and the heart rate to increase by 15 - 20 beats per minute in normotensive individuals. These responses are exacerbated in hypertensive patients in whom the SBP can increase by 90 mmHg and heart rate by 40 beats per minute [122]. Following intubation and maintenance of anesthesia, the mean arterial pressure tends to fall due to a variety of factors. Some of these factors include inhibition of the sympathetic nervous system, and loss of the baro-receptor reflex control of blood pressure. Patients with preexisting hypertension are more likely to experience intraoperative labile blood pressure [84]. Blood pressure and heart rate increase during emergence from anesthesia and during the immediate postoperative period. Hypertensive individuals in particular may experience significant increases in these parameters [123]. These rapid hemodynamic changes in the presence of ISH exacerbate any potential imbalance of myocardial oxygen supply and demand [124]. As mentioned previously, the presence of LVH and DD secondary to ISH may compromise coronary perfusion by decreasing the difference between DBP and LVEDP.

### Perioperative ISH as a Cardiac Risk Factor in Low-Risk Patients

Perioperative cardiac complications frequently complicate non-cardiac surgery patients. In 1990, perioperative cardiac complications affected up to 1 million patients and incurred an estimated cost of US$20 billion [125]. When a cardiac complication occurs, the length of stay on average is increased by 11 days [126]. With the aging population, the disease burden from cardiac complications is expected to increase. Many of the perioperative studies focused on developing scores to identify patients that are at high risk of cardiac complications preoperatively.

A recent database study of over 700,000 patients showed that β-blockers in Revised Cardiac Risk Index (RCRI) Score 0 or 1 may increase all-cause mortality [127]. Of the denominator, RCRI Score 0 and 1 comprised 569,581 of the 663,635 eligible patients while RCRI Score ≥ 3 comprised 17,071, with RCRI Score 2 adding another 76,983 patients. Hence, RCRI Score ≥ 2 represented <15% of the total population. Of the total mortality, however, 8,443 were from RCRI Score 0 and 1 patients; only 545 were from RCRI Score ≥ 3 and 1,793 were RCRI Score 2 patients. The bulk of the burden of illness, in terms of mortality, is in the “low risk” patients by the sheer size of the denominator. This highlights that the mortality occurred in low risk patients with the majority of death occurred in “low risk” group. This may be due to low risk patients having idiosyncratic events perioperatively, or due to risk factors that are not currently understood, or both.

### Perioperative Management of ISH

The aim of preoperative assessment is to evaluate and optimize the patient’s condition perioperatively thus minimizing adverse events or last-minute surgery cancellation. Evaluation of ISH patients presenting for surgery should follow the standard preoperative steps in obtaining medical history, physical examination and investigations. Acute onset of sever hypertension raises the suspicion of a secondary form of ISH. Patients with a history of cardiac, vascular or renal disease should be ensured about the level of their hypertension control. Individuals may have undiagnosed ISH for years without having had their blood pressure checked. Therefore, a careful history of end organ damage should be obtained. History of other cardiac risk factors including hypercholesterolemia, diabetes mellitus, tobacco, and family history of coronary artery disease should be obtained. If a secondary form of ISH is suspected, details of possible causes should be explored including renal diseases, pheochromocytoma, hyperthyroidism, and coarctation of aorta. An accurate measurement of blood pressure with a proper cuff size is crucial. The patient should rest quietly in the pre-assessment unit for at least five minutes before the blood pressure measurement. Cardiovascular examination should focus on listening for S3, S4 and murmurs of coexisting valvular disease. Peripheral pulses should also be palpated to assess for vascular disease. Also, ausculatation for renal artery bruit over the upper abdomen: the presence of a unilateral bruit with both a systolic and diastolic component suggests renal artery stenosis. A funduscopic evaluation of the eyes should be performed to detect any evidence of hypertensive retinopathy. Investigations in relation to ISH should be directed toward assessing the extent of the end organ damage.

Currently, there is no evidence to identify stage 1 diastolic hypertension (DBP >90 - < 110 mmHg) as a predictor of adverse perioperative outcomes [88,89]. When DBP is ≥ 110mmHg, the traditional approach is to defer elective surgery [128]. This practice is not based on level 1 evidence, but solely on the perception that such elevated pressure is associated with increased perioperative risk [129]. 

In patients with ISH undergoing CABG, there is evidence that the incidence of perioperative cardiovascular complications is increased [129]. Also, the incidence of postoperative myocardial ischemia has been shown to increase with increasing admission SBP [94]. This may be of concern since the association between postoperative myocardial ischemia and postoperative ischemic cardiac events has been shown to be significant [130-132]. Induction of anesthesia in elderly patients with uncontrolled SBP showed substantial decrease of systolic pressure and stroke volume with marked vasopressor response to noxious stimuli [124]. Tamborini *et al.* [133], noted not only the significant reduction of systemic blood pressure in the uncontrolled ISH but also a decrease in the coronary blood flow during induction of anesthesia. In addition, high blood pressure was found to be a major risk factor for coronary artery disease [67], congestive heart failure [134], renal, and cerebrovascular diseases [135]. Any of these factors increase the likelihood of perioperative myocardial infarction or death [136]. In the absence of strong evidence, individual physicians would have to make their own decisions regarding ISH and the level SBP to warrant surgical cancellation. Nevertheless, it seems appropriate to defer elective surgery where possible in patients with stage 2 ISH (SBP ≥ 180), especially if there is evidence of target organ damage.

## CONCLUSION

In the community, ISH is the most prevalent subtype of hypertension and is a significant predictor for cardiovascular complications. Nevertheless, it is not recognized as a significant cardiac risk factor in the surgical setting. A number of reasons may explain why ISH is an unrecognized cardiovascular risk factor in the perioperative period. In addition, pathophysiological changes such as LVH and diastolic dysfunction as a result of ISH, in conjunction with the sympathetic and stress hormonal surges at the time of anesthesia emergence as well as during the postoperative period, provide the rationale that perioperative ISH deserves further studies.

Information emerging from a large database suggested that majority of the perioperative mortalities occurred in low-risk groups. This attracted and will attract the attentions of clinical investigators to look at possible under diagnosed risk factors. We believe that ISH is under diagnosed risk factors. Because of the lack of perioperative ISH clinical trials, we were not able to conduct a systematic review or a meta-analysis of perioperative ISH at this stage.

Until we have the complete necessary data, we will continue using best practice based on the available data. The current work of the PROMISE study will be the stepping-stone toward answering the question; is the perioperative ISH a cardiac risk factor?

## Figures and Tables

**Fig. (1):**
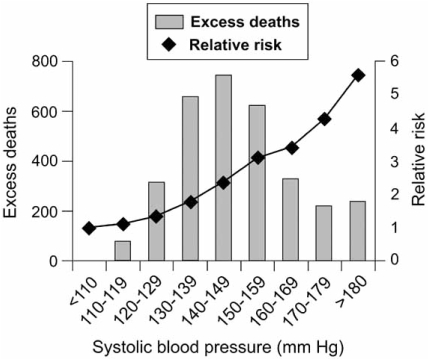
The effect of systolic pressure at entry to MRFIT (Multiple Risk Factor Intervention Trial) on the relative risk of death because of coronary artery disease. The number of excess deaths is calculated relative to the number of deaths because of coronary artery disease that would be expected from the death rate in the baseline group, that is those patients with a systolic arterial pressure of less than 110 mm Hg.^3^ Permission from Oxford University Press (BJA, 2004; 92: 570-83).

**Fig. (2):**
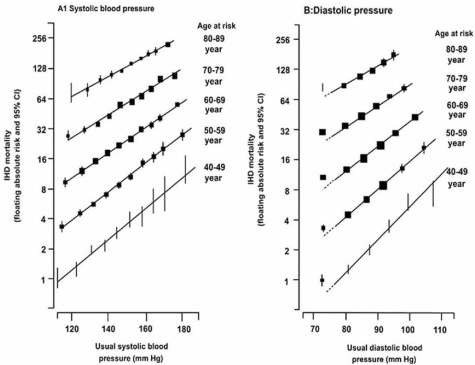
Ischemic heart disease (IHD) mortality rate in each decade of age versus usual blood pressure at the start of that decade. Source: Permission from the Author (*The Lancet*, 2002; 360: 1903–1913).

**Fig. (3):**
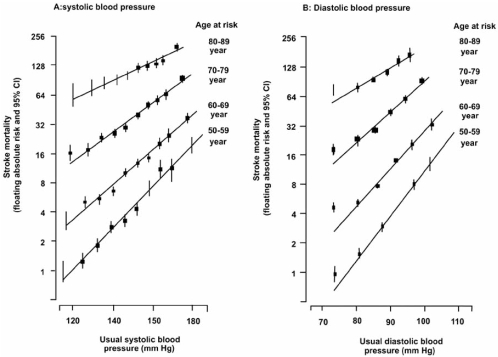
Stroke mortality rate in each decade of age versus usual blood pressure at the start of that decade. Source: Reprinted with permission from the Author (*The Lancet*, 2002; 360: 1903–1913).

**Fig. (4):**
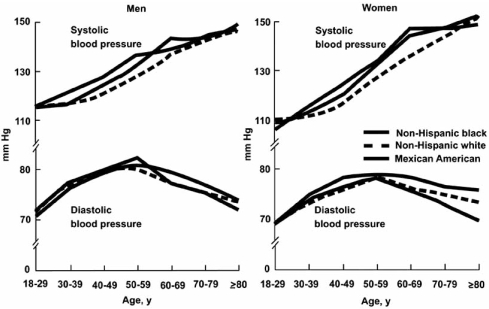
Changes in systolic and diastolic blood pressure with age. SBP and DBP by age and race or ethnicity for men and women over 18 years of age in the US population. Data from NHANES III, 1988 to 1991. Permission source: Roccella EJ et al. Seventh Report of the Joint National Committee on Prevention, Detection, Evaluation, and Treatment of High Blood  Pressure *Hypertension* 2003; 42: 1206.

**Table 1.** Classification of Hypertension as Identified by Joint National Committee –VII (JNC-VII) [13]

BP Classification	SPB* mmHg	DBP* mmHg	Lifestyle Modification	Initial Drug Therapy	
				Without compelling Indication	With Compelling Indication
**Normal**	< 120	And <80	Encourage	No antihypertensive Drug indicated	Drug(s) for compelling indications⇟
**Pre-hypertension**	120-139	Or 80-90	Yes		
**Stage 1 Hypertension**	140-159	Or 90-99	Yes	Thiazide-type diuretics for most, May consider ACEI, ARB, BB, CCB, or combination	Drug(s) for the compelling indications. Other antihypertensive drugs (diuretics, ACEI, ARB, BB, CCB) as needed
**Stage 2 Hypertension**	≥160	Or≥100	Yes	Two-drug combination for most⇞ (usually thiazide-type diuretic and ACEI or ARB or BB or CCB	

DBP, diastolic blood pressure; SBP, systolic blood pressure

Drug abbreviations: ACEI, angiotensine converting enzyme inhibitor; ARB, angiotensin receptor blocker; BB; beta-blocker; CCB, calcium channel blocker.

. Treatment determined by highest BP category

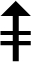
 Initial combined therapy should be used cautiously in those at risk for orthostatic hypotension

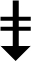
 Treat patients with chronic kidney disease or diabetes to BP goal of < 130/80 mmHg
